# Interstitial fibrosis and arrhythmic mitral valve prolapse: Unraveling sex-based differences

**DOI:** 10.1016/j.jocmr.2024.101117

**Published:** 2024-10-28

**Authors:** Lionel Tastet, Shalini Dixit, Rohit Jhawar, Thuy Nguyen, Mohammad Al-Akchar, Dwight Bibby, Farzin Arya, Luca Cristin, Shafkat Anwar, Satoshi Higuchi, Henry Hsia, Yoo Jin Lee, Francesca N. Delling

**Affiliations:** a Department of Medicine (Cardiovascular Division), University of California, San Francisco, California, USA; b Carle Illinois School of Medicine, University of Illinois-Urbana Champaign, Champaign, Illinois, USA; c Department of Pediatrics, Division of Cardiology, University of California, San Francisco, California, USA; d Department of Electrophysiology, Division of Cardiology, University of California, San Francisco, California, USA; e Department of Radiology, Division of Cardiology, University of California, San Francisco, California, USA

**Keywords:** Mitral valve prolapse, Interstitial myocardial fibrosis, Arrhythmia, Sex differences

## Abstract

**Background:**

Interstitial fibrosis as quantified by cardiovascular magnetic resonance (CMR) has been demonstrated in arrhythmic mitral valve prolapse (AMVP), a condition with known female predominance. Prior studies of interstitial fibrosis in AMVP have only included cases with significant mitral regurgitation (MR) or mitral annular disjunction (MAD), limiting our understanding of alternative arrhythmic mechanisms in mitral valve prolapse (MVP). We sought to evaluate the association between interstitial fibrosis and AMVP, regardless of MAD and without severe MR, while also investigating the contribution of sex to this association.

**Methods:**

We performed research-based contrast CMR in consecutive individuals with MVP between 2019 and 2022. Extracellular volume fraction (ECV%), a surrogate marker for interstitial fibrosis, was quantified using T_1_ mapping in the basal and mid-left ventricular slices. Replacement fibrosis was assessed using late gadolinium enhancement (LGE). AMVP was defined as MVP with frequent premature ventricular contractions and/or non-sustained/sustained ventricular tachycardia (VT) or fibrillation (VF).

**Results:**

We identified 65 MVP cases without severe MR (30 [46%] women, 22 [34%] no/trace, 30 [44%] mild, and 13 [21%] moderate MR) and with adequate ECV% measurement. Among these, 38% were classified as AMVP, including two cases of aborted VF arrest, both in premenopausal females. Global ECV% was significantly higher in AMVP vs non-AMVP (31% [27-33] vs 27% [23-30], p = 0.002). In the AMVP group, higher segmental ECV% was not limited to the inferolateral/inferior walls, typically subject to myocardial traction by the prolapsing leaflets/MAD but was more diffuse and involved atypical segments such as the anterior/anterolateral walls (p < 0.05). The association between AMVP and global ECV% was driven by female sex (32% [30-34] vs 27% [25-30], p = 0.002 in females; 28% [23-32] vs 26% [23-30], p = 0.41 in males). ECV% remained independently associated with an increased risk of arrhythmic events, including VT/VF (p < 0.01), even after adjustment for cardiovascular risk factors, MAD, and LGE (p < 0.01).

**Conclusion:**

In MVP without significant MR, interstitial fibrosis by CMR is associated with an increased risk of arrhythmic events, suggesting a primary myopathic process. The selective association between interstitial fibrosis and AMVP in females may explain why severe arrhythmic complications are more prevalent among women.

## Graphical abstract

**Figure:**
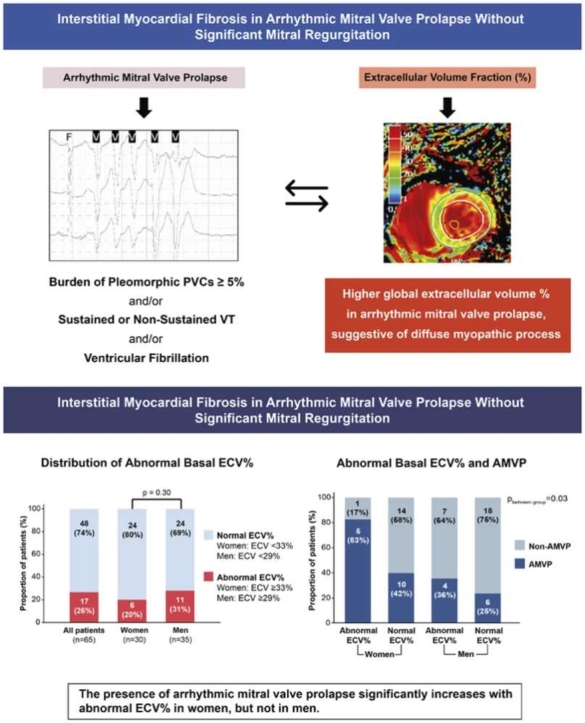
Expansion of interstitial myocardial fibrosis in arrhythmic mitral valve prolapse. Association between arrhythmic mitral valve prolapse (AMVP) and interstitial fibrosis as demonstrated by T_1_ mapping on CMR (Top). Abnormal extent of ECV% relates to more cases of AMVP in women, but not in men (Bottom). *MR* mitral regurgitation, *PVC* premature ventricular contraction, *VT* ventricular tachycardia

## 1 Introduction

Mitral valve prolapse (MVP) is a common heritable condition affecting 2% to 3% of individuals worldwide [1]. MVP without significant mitral regurgitation (MR) is generally considered a benign condition. However, a small but non-negligible subset of MVP subjects (0.14% to 1.8% yearly) are at risk of life-threatening arrhythmic events and sudden cardiac death (SCD) [2], [3]. This “malignant” form of MVP has been commonly associated with female sex, younger age, bileaflet involvement, replacement fibrosis in the papillary muscles/adjacent left ventricular (LV) wall, and frequent or complex ventricular ectopy [3], [4]. The latter may occur even in MVP without significant MR and is associated with excess mortality [4], [5], [6].

The role of myocardial fibrosis as a substrate for ventricular ectopy and SCD in MVP is supported by seminal histologic and imaging studies [7], [8], [9], [10]. Indeed, MVP-related abnormal valvular-myocardial mechanics related to greater prolapse, mitral annular disjunction (MAD), excessive displacement of papillary muscles, and curling of the basal myocardium may induce myocardial fibrosis [11]. Replacement fibrosis, typically involving the papillary muscles and LV inferolateral wall, can be detected by late gadolinium enhancement (LGE) on cardiovascular magnetic resonance (CMR) and relates to arrhythmic MVP (AMVP) [8], [12], [13]. However, severe arrhythmic events may occur even in the absence of replacement myocardial fibrosis [9], [14]. In addition to LGE, CMR can detect diffuse or interstitial fibrosis using the extracellular volume fraction (ECV%) calculated from T_1_ mapping [15]. As shown by our group in a post-mortem investigation [16], diffuse interstitial myocardial fibrosis could represent an alternative substrate for life-threatening arrhythmias in MVP [17], regardless of MR severity or replacement fibrosis. Prior studies investigating the role of T_1_ mapping/ECV% in AMVP have included mostly cases with severe MR, limiting our understanding of a primary myopathic process in MVP [17], [18]. Furthermore, it remains uncertain whether sex-related differences, which may impact not only the mitral valve (MV) apparatus, but also myocardial structure and function, could influence the contribution of diffuse myocardial fibrosis to arrhythmic events in MVP [19].

We therefore hypothesized that among individuals with AMVP and without significant MR: 1) interstitial fibrosis as quantified by global ECV% on CMR is increased, suggesting a primary myopathy; and 2) the relationship between interstitial fibrosis and ventricular arrhythmias is influenced by female sex.

## 2 Methods

### 2.1 Study population

**Fig. 1:**
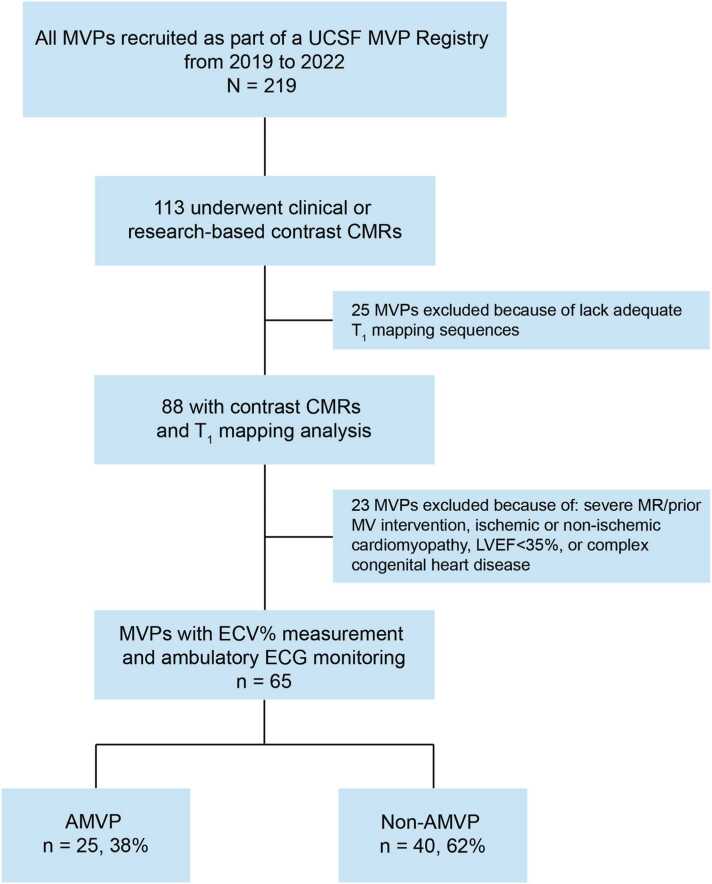
Study flowchart. *CMR* cardiac magnetic resonance, *AMVP* arrhythmic mitral valve prolapse, *ECG* electrocardiogram, *ECV* extracellular volume, *ECV%* ECV fraction, *MR* mitral regurgitation, *MV* mitral valve, *MVP* mitral valve prolapse, *UCSF* University of California San Francisco

Patients with MVP who presented to the University of California San Francisco (UCSF) for clinical care between 2019 and 2022 were recruited as part of the UCSF MVP Registry. A total of 219 patients consented, of which 88 underwent contrast CMRs with T_1_ mapping (Fig. 1). We then excluded cases with 1) severe MR and/or prior MV intervention; 2) ischemic or non-ischemic cardiomyopathy (including sarcoidosis); 3) LV ejection fraction (LVEF) <35%; 4) complex congenital heart disease; 5) >mild aortic valve disease; 6) defibrillator implantation before CMR examination; or 7) lack of comprehensive ambulatory electrocardiogram monitoring. All remaining 65 MVPs had complete demographic/clinical variables within a calculated Charlson comorbidity index [20], laboratory, rhythmic, and CMR data.

### 2.2 Ascertainment of ventricular arrhythmias

Ventricular arrhythmias were ascertained through 48-hour Holter monitors obtained on the same day of the CMR study. The definition of AMVP followed the criteria outlined in the expert consensus statement from the European Heart Rhythm Association [6], [21]. AMVP was defined as follows: either frequent (≥5%) premature ventricular contractions (PVCs) (isolated and/or in couplets/triplets) or complex ventricular ectopy, including sustained or non-sustained ventricular tachycardia (NSVT) or ventricular fibrillation (VF) [6]. Arrhythmic cause of death was ascertained using the National Death Index for SCD cases. PVC burden and origin (i.e., papillary muscles, MV/annulus, LV/RV outflow tract, or other) were also recorded. Pleomorphic ventricular ectopy was defined as PVCs of at least two different morphologies. NSVT was defined as >3 PVCs with a rate >100 bpm lasting <30 seconds [6], [14].

### 2.3 Cardiac magnetic resonance imaging

#### 2.3.1 Acquisition

Imaging was performed using the UCSF Core using 3.0T scanners Discovery MR750w or SIGNA Premier (GE Healthcare, Milwaukee, Wisconsin, USA) [22]. Briefly, standard long- and short-axis cine images covering the entire LV were obtained using a balanced steady-state free precession (SSFP) sequence. Typical parameters were repetition time/echo time 2.8/1.3 ms, flip angle 45º. LGE was performed 5-15 minutes following injection of 0.1 mmol/kg gadobutrol, and images were obtained using a high-resolution breath-hold two-dimensional (2D) sequence at three separate levels in the short-axis plane (basal, mid, and apical). An inversion-recovery fast gradient-echo sequence was performed in two phase-encoding directions to differentiate true late enhancement from artifact [23]. The inversion time was optimized to achieve satisfactory nulling of the myocardium. T_1_ mapping data were acquired using a Modified Look-Locker Inversion-recovery sequence with built-in motion correction and an acquisition scheme of 3(3)-3(3)-5 both before and following contrast agent administration. A 2D phase-contrast (through-plane), velocity-encoded, imaging approach through the ascending aorta was utilized to quantify MR [24]. When quantitative assessment of MR severity was not available, MR jets were assessed qualitatively using the extent of signal loss due to spin dephasing on SSFP imaging [24].

#### 2.3.2 Image analysis

Image analysis was performed offline using a standardized approach (cvi42 version 5.14.0, Circle Cardiovascular Imaging Inc., Calgary, Alberta, Canada). LV volume and mass were indexed to body surface area. Papillary muscles were included when measuring LV mass and excluded when measuring volumes [23]. MVP was defined as superior displacement of one or both mitral leaflets >2 mm beyond the mitral annulus on long-axis view, as previously described [7]. The MVP phenotype was further subdivided as bileaflet or single-leaflet (anterior or posterior). MR volume was calculated as the difference between LV stroke volume and the forward aortic flow volume. MR fraction was obtained by dividing MR volume by the LV stroke volume. The grading of MR severity using MR fraction was as follows: 1) mild ≥20%; 2) moderate 21%-39%; or severe ≥40%.

The presence and location (inferolateral segment vs other segments) of MAD, defined as a distinct separation between the MV junction, left atrial wall, and the basal segment of the LV myocardium during systole, was assessed on cine long-axis views as previously described [25]. In addition, LV “curling”, defined as an abnormal or exaggerated basal myocardial systolic motion, was qualitatively and quantitatively assessed [11], [12].

On dedicated images, LGE was recorded and quantified using a the full-width-at-half-maximum technique [23]. Areas of inversion artifact or signal contamination by epicardial fat or blood pool were manually excluded. LGE quantification was expressed as percentage of LV mass.

T_1_ mapping data were generated from a short-axis cine stack of the basal and mid-LV, and the regions of the endocardium and epicardium on the native T1 maps were manually contoured to avoid partial-volume or cardiac-motion-related blurring of the myocardium-blood boundary, where blood signal may contaminate myocardial data. A 10% offset was also applied to avoid signal contamination from the blood pool and epicardial fat (Supplemental Fig. 1). This approach is consistent with previous studies [15], [26] and ensures accurate sampling of the myocardium by excluding areas close to the endocardium and epicardium, which are non-myocardial tissues. The pre-contrast contours were then copied onto the corresponding postcontrast maps with minor adjustments made to avoid partial-volume effects. Regions of interest were then drawn in the blood, avoiding myocardium and papillary muscles. None of the patients had evidence of myocarditis, cardiac amyloidosis, or conditions related to edema based on clinical history, cardiac biomarkers, or available imaging. ECV fraction was calculated according to the following formula: ECV% = partition coefficient × [1 − hematocrit], where partition coefficient = [∆R1_myocardium_/∆R1_blood-pool_] and ∆R1 = (1/postcontrast T1 − 1/native T1) [27]. Hematocrit was measured as per protocol immediately before CMR study. Native myocardial T1 and ECV% were also recorded for all myocardial segments of the basal and mid-LV slice, including the LV inferolateral wall, a location typically subject to abnormal traction by the prolapsing leaflets [12], [18], [28]. Segments with LGE were excluded from the T1/ECV% measurement. Sex-specific thresholds of abnormal ECV% were derived from previously reported normative values in healthy volunteers, using the 95% upper limit thresholds (i.e., mean + 2 standard deviations [SD]) [29].

### 2.4 Statistical analysis

Continuous variables were expressed as mean ± SD or median (interquartile range) and were tested for normality of distribution and homogeneity of variances with the Shapiro-Wilk and Levene tests, respectively. Continuous variables were compared between groups with Student’s t test or Wilcoxon-Mann-Whitney test with Bonferroni correction for multiple comparisons, as appropriate. Categorical variables were presented as frequencies and percentages and were compared with χ^2^ test or Fisher’s exact test as appropriate. Univariable and multivariable logistic regression analyses were performed to identify the factors significantly associated with the risk of AMVP and to assess the interaction between sex and ECV% in relation to AMVP risk. The multivariable model included all clinically relevant factors (i.e., Charlson comorbidity index [20], MV morpho-functional parameters, LVEF, LGE, and ECV%). Results were presented as odds ratio (OR) with 95% confidence interval (CI). Univariable and multivariable linear regression analyses were performed to identify the associated factors of ECV%. The multivariable models included clinically relevant factors, including MV morpho-functional parameters, and those with a p value ≤0.20 in univariable analysis. Results were presented as standardized regression coefficient ± standard error.

A two-tailed p value ≤0.05 was considered significant. Statistical analyses were performed with Stata software version 14.2 (StataCorp, College Station, Texas, USA).

## 3 Results

### 3.1 Study population

Demographic, arrhythmic, and CMR characteristics of the study population are presented in Table 1. Of the 65 MVPs included in this analysis, a total of 25 (38%) cases (15 [60%] women) were AMVPs, a proportion similar to that reported in other MVP samples [11], [30], [31]. Among the AMVP patients in our cohort, two experienced aborted VF arrest and were treated with implantable cardiac defibrillators (ICDs). Both were premenopausal females who underwent CMR examination 3 days after the arrhythmic event in one case and 7 days in the other, before ICD implantation. The main characteristics of these cases are presented in Supplemental Table 1.

**Table 1:** Clinical and arrhythmic characteristics according to arrhythmic mitral valve prolapse.

	All patients (N = 65)	AMVP (n = 25; 38%)	Non-AMVP (n = 40; 62%)	p value
Clinical data				
Age, years	55 ± 15	57 ± 15	54 ± 15	0.40
Female sex, n (%)	30 (46)	15 (60)	15 (38)	0.07
Body surface area, m²	1.83 ± 0.18	1.82 ± 0.20	1.83 ± 0.17	0.76
Symptoms, n (%)	20 (31)	10 (40)	10 (25)	0.20
Dyspnea, n (%)	2 (3)	2 (8)	0 (0)	0.14
Chest pain, n (%)	5 (8)	3 (12)	2 (5)	0.37
Palpitations, n (%)	10 (15)	4 (16)	6 (15)	0.91
Syncope, n (%)	6 (9)	3 (12)	3 (8)	0.67
Charlson comorbidity index	1 (0- 3)	2 (0-4)	1 (0-3)	0.17
Beta blockers, n (%)	14 (9)	11 (44)	4 (10)	**0.002**
Antiarrhythmics, n (%)	1 (2)	1 (4)	0 (0)	0.38
Arrhythmic data				
Pleomorphic ventricular ectopy, n (%)	36 (56)	16 (67)	20 (50)	0.19
Non-sustained VT, n (%)	21 (34)	21 (91)	0 (0)	**<0.001**
PVCs burden, (%)	0.2 (0.01-1.0)	0.7 (0.1-8.9)	0.01 (0-0.5)	**<0.001**
PVCs burden ≥5%, n (%)	6 (10)	6 (25)	0 (0)	**0.002**
PVCs by origin				**0.04**
Posterior papillary muscles, n (%)	11 (23)	7 (41)	4 (13)	
Anterior papillary muscles, n (%)	9 (19)	2 (12)	7 (23)	
Anterior/posterior MV, n (%)	2 (5)	2 (12)	0 (0)	
LV/RV outflow tract, n (%)	8 (17)	2 (12)	6 (19)	
Other, n (%)	18 (38)	4 (24)	14 (45)	

Values are mean ± SD or median (interquartile range)

*AMVP* arrhythmic mitral valve prolapse, *LV* left ventricle, *MV* mitral valve, *PVC* premature ventricular contraction, *RV* right ventricle, *SD* standard deviation, *VT* ventricular tachycardia

Bold p-values indicate statistical significance.

PVC burden was significantly higher in the AMVP group, and by definition all cases in this group had PVC burden ≥5% (Table 1). PVCs were pleomorphic in all cases with PVCs burden ≥5%, and originated from typical MVP sites such as the papillary muscles and MV annulus [32] (Table 1).

### 3.2 Morpho-functional characteristics, myocardial fibrosis, and ventricular arrhythmias

Table 2 summarizes CMR characteristics according to AMVP. Quantitative assessment of MR severity was available for 46 (71%) MVP cases. Among the 19 with qualitative MR assessment using SSFP imaging, one had moderate MR and the remaining had mild MR. In the overall study sample of 65 MVPs, 30 (46%) were classified as mild and 13 (20%) as moderate MR; the remaining cases had no or trace MR. According to AMVP vs non-AMVP, MR severity (no/trace, mild, or moderate), LV volumes, and presence/location of MAD or LGE were comparable between groups (Table 2). However, the AMVP group had significantly lower LVEF (59 ± 8% vs 63 ± 6%, p = 0.02), a trend toward a higher proportion of bileaflet involvement (76% vs 55%, p = 0.08), and longer MAD (7.1 ± 2.4 vs 5.6 ± 2.2, p = 0.07), albeit not statistically significant (Table 2).

**Table 2:** CMR characteristics according to arrhythmic mitral valve prolapse.

	All patients (N = 65)	AMVP (n = 25; 38%)	Non-AMVP (n = 40; 62%)	p value
Bileaflet MVP, n (%)	41 (63)	19 (76)	22 (55)	0.08
MR fraction, %	21 (14-27)	19 (14-27)	24 (14-39)	0.72
MR severity,				0.70
No or trace, n (%)	22 (34)	7 (28)	15 (38)	
Mild, n (%)	30 (46)	13 (52)	17 (43)	
Moderate, n (%)	13 (20)	5 (20)	8 (20)	
MAD inferolateral, n (%)	34 (53)	14 (56)	20 (51)	0.71
MAD inferolateral, mm	6.3 ± 2.4	7.1 ± 2.4	5.6 ± 2.2	0.07
LV end-diastolic volume index, mL/m²	83 ± 18	82 ± 16	83 ± 19	0.91
LV end-systolic volume index, mL/m²	32 ± 10	34 ± 10	31 ± 10	0.29
LV ejection fraction, %	61 ± 7	59 ± 8	63 ± 6	**0.02**
LV mass index, g/m^2^	47 ± 15	49 ± 11	46 ± 17	0.43
LV systolic curling, n (%)	40 (65)	18 (75)	22 (59)	0.21
LV systolic curling, mm	3.1 (2.6-4.4)	3.3 (2.8-5.4)	2.9 (2.4-3.9)	0.28
LGE, n (%)	22 (33)	10 (40)	12 (30)	0.41
MVP-LGE				
Basal inferolateral LV wall, n (%)	12 (18)	5 (20)	7 (18)	0.80
Mid inferolateral LV wall, n (%)	1 (2)	0 (0)	1 (3)	0.99
Papillary muscles, n (%)	6 (9)	3 (12)	3 (8)	0.67
LGE size, %	2.0 (0.6-3.6)	2.0 (0.6-4.0)	1.9 (0.5-3.0)	0.43
T_1_ mapping				
Hematocrit, %	43 (40-45)	42 (40-45)	44 (40-45)	0.58
Basal LV native T_1_, ms	1272 (1218-1310)	1289 (1232-1331)	1264 (1218-1295)	0.23
Basal LV postcontrast T_1_, ms	571 (527-631)	547 (520-622)	573 (542-641)	0.17
Basal LV ECV, %	28 (24-31)	31 (27-33)	27 (24-30)	**0.003**
Mid-LV native T_1_, ms	1258 (1228-1289)	1275 (1247-1307)	1255 (1216-1277)	**0.01**
Mid-LV postcontrast T_1_, ms	572 (525-602)	546 (509-600)	583 (537-620)	0.13
Mid-LV ECV, %	28 (25-32)	31 (27-33)	27 (23-30)	**0.002**
Global ECV,* %	28 (25-32)	31 (27-33)	27 (23-30)	**0.002**

Values are mean ± SD or median (interquartile range)

*AMVP* arrhythmic mitral valve prolapse, *CMR* cardiac magnetic resonance, *ECV* extracellular volume, *ECV%* extracellular volume fraction, *LGE* late gadolinium enhancement, *LV* left ventricular, *MAD* mitral annular disjunction, *MR* mitral regurgitation, *MVP* mitral valve prolapse, *SD* standard deviation

Bold p-values indicate statistical significance.

* Includes ECV% of the basal and mid-LV slices

There were no significant differences in the presence or size of LGE between AMVP and non-AMVP (Table 2). While basal native and postcontrast T_1_ values were similar between groups, AMVP cases had significantly higher mid-LV native T_1_ (p = 0.01), but no significant difference in postcontrast T_1_ (Table 2). The AMVP group had significantly higher basal (p = 0.003), mid-LV (p = 0.002), and global (i.e., combination of basal and mid-LV; p = 0.002) ECV% compared to non-AMVP (Table 2).

### 3.3 Ventricular arrhythmias, diffuse interstitial fibrosis, and sex-based differences

**Fig. 2:**
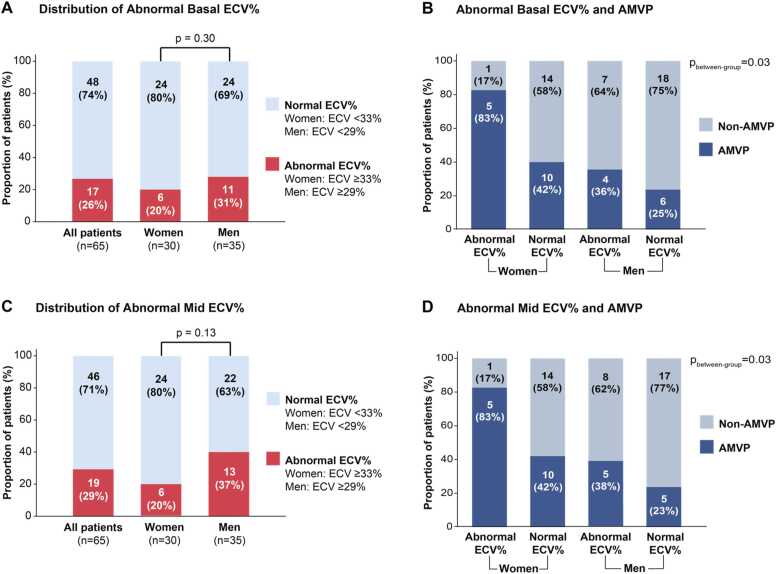
Association of extracellular volume fraction with arrhythmic mitral valve prolapse and sex-related differences. Comparison of abnormal basal ECV% (ECV ≥33% in women and ≥29% in men, based on normal values [29]) in the entire population and according to sex (A). Proportion of abnormal basal ECV% according to AMVP and sex (B). Comparison of abnormal mid-LV ECV% (ECV ≥33% in women and ≥29% in men) in the entire population and according to sex (C). Proportion of abnormal mid-LV ECV% according to AMVP and sex (D). Numbers and (%) of individuals in each category are presented in the bar graphs. The numbers in brackets below each bar graph indicate the total number of patients for each subgroup. *AMVP* arrhythmic mitral valve prolapse, *ECV* extracellular volume, *ECV%* ECV fraction, *LV* left ventricular

When we considered the interaction between sex and ECV% in determining risk of ventricular arrhythmias, there was a trend toward a stronger association between ECV% and AMVP in women compared to men (mid-ECV% p = 0.07). Using sex-specific thresholds of abnormal ECV% (i.e., ECV ≥33% in women and ≥29% in men, based on normal values [29]), a greater proportion of AMVP cases were observed with abnormal ECV% in women (Fig. 2). This pattern was consistent both for basal (Fig. 2A and B) and mid (Fig. 2C and D) ECV%.

**Fig. 3:**
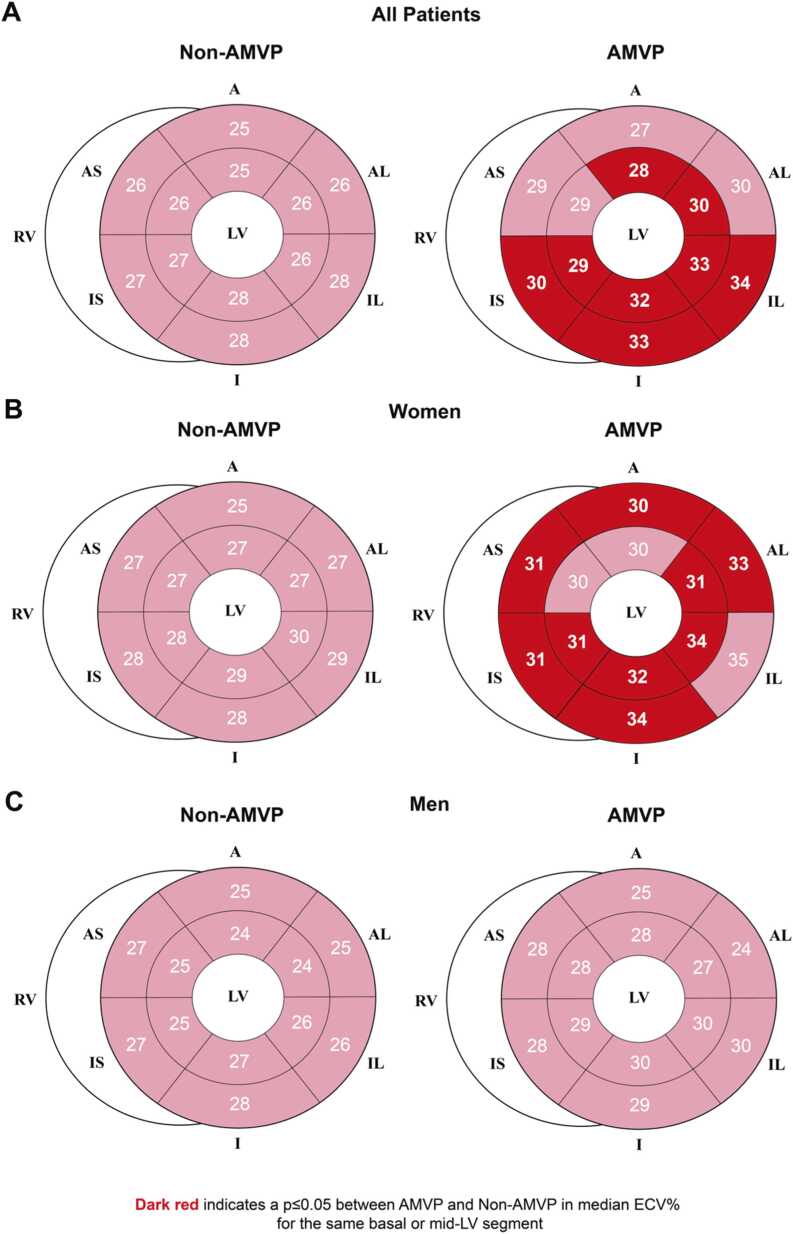
Distribution of extracellular volume fraction in myocardial segments according to arrhythmic mitral valve prolapse. Comparison of median ECV% in the basal and mid-LV myocardial segments according to arrhythmic mitral valve prolapse in the whole cohort (A), subset of women (B), and men (C). *A* anterior, *AL* anterolateral, *AMVP* arrhythmic mitral valve prolapse, *AS* anteroseptal, ECV% extracellular volume fraction, *I* inferior, *IL* inferolateral, *IS* inferoseptal, *LV* left ventricular

Fig. 3 shows the distribution of ECV% across basal and mid-LV segments. Compared to non-AMVP, AMVP demonstrated higher ECV% in atypical segments such as the anterior/anterolateral segments (p ≤ 0.05) in addition to the inferolateral/inferior LV, more typical areas of myocardial traction by the prolapsing leaflet and/or MAD (Fig. 3A). Women with AMVP had higher segmental ECV% across most segments compared to women without AMVP (all p ≤ 0.05) (Fig. 3B). However, an equivalent diffuse pattern of abnormal ECV% was not observed in men with AMVP, who had similar median segmental ECV% values compared to non-AMVP (Fig. 3C). Furthermore, among men with AMVP, the inferior/lateral segments exhibited higher ECV% compared to the other segments (Fig. 3C).

Table 3 summarizes CMR characteristics by AMVP and sex. Among women, mid-LV native T_1_ was significantly higher in AMVP cases (median: 1285 [1250-1343] vs 1270 [1234-1277] ms, p = 0.02). Women with AMVP were also more likely to have bileaflet MVP, LV systolic curling, and lower LVEF (Table 3). In men, only LVEF was significantly lower in AMVP vs non-AMVP (56 ± 9% vs 62 ± 6%, p = 0.04). MR severity, inferolateral MAD, and LGE did not differ between groups among women and men (Table 3).

**Table 3:** CMR Related characteristics according to sex and arrhythmic mitral valve prolapse.

	Women			Men		
	AMVP (n = 15; 50%)	Non-AMVP (n = 15; 50%)	p value	AMVP (n = 10; 29%)	Non-AMVP (n = 25; 71%)	p value
Age, years	54 ± 15	50 ± 16	0.48	60 ± 16	55 ± 14	0.35
Heart rate, bpm	66 ± 8	64 ± 11	0.62	66 ± 13	58 ± 13	0.11
Bileaflet MVP, n (%)	11 (73)	6 (43)	0.06	8 (80)	16 (64)	0.35
MR severity, n (%)			0.54			1.00
No or trace, n (%)	4 (29)	8 (53)		3 (30)	6 (27)	
Mild, n (%)	7 (50)	5 (33)		5 (50)	10 (45)	
Moderate, n (%)	3 (21)	2 (13)		2 (20)	6 (27)	
MAD inferolateral, n (%)	9 (60)	8 (57)	0.87	5 (50)	12 (48)	0.91
LV end-diastolic volume index, mL/m^2^	79 ± 14	80 ± 18	0.90	87 ± 19	85 ± 20	0.75
LV end-systolic volume index, mL/m^2^	31 ± 7	29 ± 8	0.42	38 ± 12	33 ± 10	0.19
LV ejection fraction, %	60 ± 6	64 ± 5	0.08	56 ± 9	62 ± 6	**0.04**
LV mass index, g/m^2^	44 ± 8	40 ± 11	0.26	54 ± 11	49 ± 19	0.39
LV systolic curling, n (%)	12 (86)	8 (57)	0.09	6 (60)	14 (61)	0.96
LV systolic curling, mm	3.4 (2.9-5.7)	2.9 (2.1-4.7)	0.31	3.0 (2.7-3.4)	2.8 (2.6-3.6)	0.95
LGE, n (%)	4 (27)	4 (27)	0.99	8 (60)	6 (32)	0.15
MVP-LGE						
Basal inferolateral LV wall, n (%)	1 (7)	2 (13)	1.00	3 (30)	4 (16)	0.38
Mid inferolateral LV wall, n (%)	0 (0)	0 (0)	-	0 (0)	1 (4)	0.52
Papillary muscles, n (%)	1 (7)	2 (13)	1.00	2 (20)	1 (4)	0.19
T_1_ mapping						
Hematocrit, %	41 (40-44)	40 (38-44)	0.43	44 (41-45)	45 (42-46)	0.39
Basal LV native T_1_, ms	1299 (1248-1337)	1264 (1218-1312)	0.24	1240 (1212-1289)	1262 (1215-1292)	0.34
Basal LV postcontrast T_1_, ms	527 (495-579)	562 (526-624)	0.38	601 (545-645)	590 (545-644)	0.89
Basal LV ECV, %	32 (30-35)	27 (25-30)	**0.003**	28 (22-31)	27 (23-30)	0.51
Mid-LV native T_1_, ms	1285 (1250-1343)	1270 (1234-1277)	**0.02**	1248 (1234-1285)	1232 (1211-1276)	0.34
Mid-LV postcontrast T_1_, ms	527 (505-600)	591 (501-636)	0.35	585 (525-602)	578 (544-610)	0.72
Mid-LV ECV, %	32 (30-33)	28 (26-30)	**0.002**	29 (25-33)	27 (23-30)	0.26
Global ECV, %	32 (30-34)	27 (25-30)	**0.002**	28 (23-32)	55 ± 14	0.41

Values are mean ± SD

*AMVP* arrhythmic mitral valve prolapse, *CMR* cardiac magnetic resonance, *ECV* extracellular volume, *LGE* late gadolinium enhancement, *LV* left ventricular, *MAD* mitral annular disjunction, *MR* mitral regurgitation, *MVP* mitral valve prolapse, *SD* standard deviation

Bold p-values indicate statistical significance.

### 3.4 Factors associated with arrhythmic MVP

In an exploratory analysis, we evaluated the association of ECV% with AMVP independently of traditional imaging risk factors, including presence of LGE, MR severity, bileaflet MVP, greater inferolateral MAD (i.e., length ≥6 mm, median of the study population), and LVEF (Table 4). In univariable analysis, both basal and mid-ECV% (OR: 1.18, 95% CI 1.04-1.34; p = 0.009 and OR: 1.18, 95% CI 1.04-1.33; p = 0.01, respectively), greater inferolateral MAD (OR: 3.73, 95% CI 1.27-10.9; p = 0.02), and LVEF (OR: 0.92, 95% CI 0.85-0.99; p = 0.03) were significant predictors of AMVP (Table 4). When stratified based on sex, basal and mid-ECV% were significantly associated with AMVP in women (OR: 1.31, 95% CI 1.04-1.64; p = 0.02 and OR: 1.46, 95% CI 1.08-1.97; p = 0.01, respectively), but not in men (OR: 1.06, 95% CI 0.90-1.25; p = 0.47 and 1.07, 95% CI 0.93-1.24; p = 0.33). After multiple nested multivariable models, ECV% remained independently associated with AMVP in all multivariable models (Table 4).

**Table 4:** Factors associated with arrhythmic mitral valve prolapse.

	Univariable analysis			Multivariable analysis			Multivariable analysis	
	OR (95% CI)	p value		OR (95% CI)	p value		OR (95% CI)	p value
Model #1								
Basal ECV (per % increase)	**1.18 (1.04-1.34)**	**0.009**		**1.20 (1.05-1.37)**	**0.007**		-	-
Mid-ECV (per % increase)	**1.18 (1.04-1.33)**	**0.01**		-	-		**1.20 (1.05-1.36)**	**0.008**
Charlson comorbidity index	1.20 (0.96-1.50)	0.11		1.23 (0.96-1.58)	0.09		1.22 (0.96-1.57)	0.10
LGE	1.55 (0.55-4.43)	0.40		1.22 (0.37-3.99)	0.73		1.21 (0.39-3.79)	0.75
Model #2								
Basal ECV (per % increase)	**1.18 (1.04-1.34)**	**0.009**		**1.20 (1.05-1.36)**	**0.007**		-	-
Mid-ECV (per % increase)	**1.18 (1.04-1.33)**	**0.01**		-	-		**1.19 (1.05-1.36)**	**0.008**
Charlson comorbidity index	1.20 (0.96-1.50)	0.11		1.24 (0.97-1.58)	0.08		1.24 (0.97-1.59)	0.08
MR severity (per grade)	1.20 (0.60-2.40)	0.61		1.11 (0.52-2.38)	0.78		1.15 (0.54-2.46)	0.72
Model #3								
Basal ECV (per % increase)	**1.18 (1.04-1.34)**	**0.009**		**1.19 (1.04-1.35)**	**0.01**		-	-
Mid-ECV (per % increase)	**1.18 (1.04-1.33)**	**0.01**		-	-		**1.18 (1.03-1.34)**	**0.01**
Charlson comorbidity index	1.20 (0.96-1.50)	0.11		1.27 (0.99-1.63)	0.05		1.28 (0.99-1.65)	0.05
Bileaflet MVP	2.59 (0.85-7.85)	0.09		2.20 (0.64-7.59)	0.21		2.54 (0.75-8.65)	0.14
Model #4								
Basal ECV (per % increase)	**1.18 (1.04-1.34)**	**0.009**		**1.20 (1.05-1.36)**	**0.007**		-	-
Mid-ECV (per % increase)	**1.18 (1.04-1.33)**	**0.01**		-	-		**1.19 (1.05-1.36)**	**0.008**
Charlson comorbidity index	1.20 (0.96-1.50)	0.11		1.24 (0.97-1.58)	0.08		1.25 (0.98-1.59)	0.07
MAD inferolateral ≥6 mm	**3.73 (1.27-10.9)**	**0.02**		3.00 (0.93-9.71)	0.07		**3.69 (1.13-12.0)**	**0.03**
Model #5								
Basal ECV (per % increase)	**1.18 (1.04-1.34)**	**0.009**		**1.18 (1.03-1.35)**	**0.02**		-	-
Mid-ECV (per % increase)	**1.18 (1.04-1.33)**	**0.01**		-	-		**1.22 (1.06-1.41)**	**0.005**
Charlson comorbidity index	1.20 (0.96-1.50)	0.11		1.23 (0.95-1.59)	0.11		1.25 (0.96-1.63)	0.09
LVEF (per % decrease)	**0.92 (0.85-0.99)**	**0.03**		0.92 (0.84-1.00)	0.06		**0.89 (0.82-0.98)**	**0.02**

Values are odds ratio with 95% confidence interval. Bold values indicate p ≤ 0.05

*CI* confidence interval, *ECV* extracellular volume fraction, *LGE* late gadolinium enhancement, *LVEF* left ventricular ejection fraction, *MAD* mitral annular disjunction, *MR* mitral regurgitation, *MVP* mitral valve prolapse, *OR* odds ratio

### 3.5 Factors associated with ECV expansion

We further explored the association of various factors with basal and mid-ECV% (Supplemental Table 2). In univariable analysis, female sex, heart rate, and AMVP were the only factors significantly associated with basal or mid-ECV% (p ≤ 0.05 for all) (Supplemental Table 2). Other clinically relevant factors, including bileaflet MVP and greater MAD, showed positive but non-significant associations with basal or mid-ECV% (p ≥ 0.15 for all) (Supplemental Table 2). Additionally, no significant association was observed between MR grade and basal ECV% (p = 0.80), or mid-ECV% (p = 0.83). Similarly, the origin of PVCs (i.e., papillary muscles, MV/annulus, or LV/RV outflow tract vs other locations) was not associated with basal (p = 0.22) nor mid-ECV% (p = 0.59) (Supplemental Table 2). After multivariable adjustment, female sex remained the only significant predictor of both basal and mid-ECV% (p ≤ 0.05 for all), while heart rate remained only associated with mid-ECV% (p = 0.01) (Supplemental Table 2). Further adjustment for the use of beta blockers or antiarrhythmics did not change the association between heart rate and mid-ECV% (β = −0.28; p = 0.02).

## 4 Discussion

In this cross-sectional analysis of consecutive MVPs with predominantly no or mild MR undergoing CMR, we investigated the relationship of interstitial myocardial fibrosis with AMVP, including cases with ventricular tachycardia, VF arrest, and SCD. The main findings were as follows: 1) diffuse interstitial fibrosis as quantified by global ECV%/T_1_ mapping was significantly greater in AMVPs, irrespective of LGE; 2) in the AMVP group, higher segmental ECV% was not limited to the inferolateral/inferior LV wall, but was also demonstrated in segments not typically subject to myocardial traction, suggesting a diffuse myopathic process in MVP beyond abnormal valvular-myocardial mechanics; 3) the association between interstitial fibrosis and AMVP was more prominent in women compared to men (Graphical Abstract).

### 4.1 Myocardial fibrosis and arrhythmic MVP

Seminal studies support the role of myocardial fibrosis, resulting from increased myocardial traction by the prolapsing leaflets as a substrate for ventricular arrhythmias and SCD in MVP [4], [7], [8], [10]. Basso et al [8] previously reported that replacement fibrosis is a common feature of young SCD victims (≤40 years of age) with AMVP. However, replacement fibrosis is not consistently found in SCD victims with MVP [9], nor in those with ventricular ectopy alone (prevalence of LGE 36%-45%) [17], [33]. These findings suggest that other myocardial substrates may be contributing to arrhythmic outcomes in MVP.

Non-invasive measurement of diffuse interstitial fibrosis can be achieved by measuring ECV% by T_1_ mapping on CMR, a technique that has been validated against histology [34], [35]. Pradella et al [36] reported significantly increased ECV%, higher native T_1_, and shorter postcontrast T_1_ times in MVP compared to controls. Previously, we also found significantly shorter postcontrast T_1_ in arrhythmic MVP, suggesting greater diffuse fibrosis; however, native T_1_ times and ECV% were not measured in that analysis [17]. In the present study, ECV% was strongly associated with AMVP risk, whereas only native T_1_ time in the mid-LV slice showed a statistically significant difference between AMVPs and non-AMVPs, with postcontrast T_1_ times being numerically shorter. The discrepancies in the predictive values of myocardial T_1_ between these studies and our study may be attributed to differences in patient populations, MR severity, CMR protocols, and magnetic field strengths. Nonetheless, our findings support the use of ECV% as a more consistent surrogate marker for diffuse interstitial fibrosis, given its consistency across various scanners and magnetic fields [15].

Prior studies investigating the role of ECV% have included predominantly MVP cases with severe MR or MAD, or have linked abnormal ECV% to a few cases of sudden cardiac arrest rather than the wider spectrum of ventricular arrhythmias.[17], [18] Frequent or complex ventricular ectopy is frequently detected (>80%) in patients with MVP before lethal events and has been linked to mortality excess [4], [5]. Therefore, the identification of MVPs at risk for ventricular arrhythmias, especially when asymptomatic, remains an essential step toward improved arrhythmic risk stratification. Selection of specific categories of MVP in prior studies (only severe MR or MVP with MAD) has limited our understanding of the distribution and pattern of interstitial fibrosis in MVP beyond a simple response to chronic volume load or a focal fibrotic response to myocardial traction. Another study has assessed ECV% in mild or moderate MR, but only in 12 MVP patients and without investigating segmental contributions to global ECV% [37]. In the present study, we demonstrate for the first time in a larger sample of CMR studies unselected for MAD and without significant MR, that AMVP is strongly linked to greater ECV%, even in the subset without LGE. Our imaging findings are in line with our prior post-mortem data demonstrating that multisite interstitial fibrosis was more prevalent than replacement fibrosis (100% vs 30%) on autopsy in consecutive and unselected MVPs with sudden arrhythmic death adjudication [9]. Therefore, diffuse interstitial fibrosis may represent an alternative substrate for ventricular arrhythmias and SCD in MVP with mild MR, even in the absence of LGE. As such, MVP patients undergoing CMR for assessment of more traditional parameters of risk such as MAD or LGE, may greatly benefit from routine T_1_ mapping/ECV% calculation as part of their clinical care.

To date, the exact mechanisms for ventricular arrhythmias and SCD in MVP without severe MR are not well understood. It has been hypothesized that the arrhythmogenic risk associated with bileaflet MVP with mild MR could be attributed to stretching forces exerted on the papillary muscles and adjacent LV wall by the prolapsing leaflets [10], [11]. These mechanical stresses could trigger myocardial inflammation and contribute to fibrosis as a substrate for ventricular arrhythmias [30], [37]. Moreover, MAD, which is more common in bileaflet MVP [30], [31], can augment such abnormal valve-myocardial mechanics. In the present study, we confirmed the regionalized expansion of myocardial fibrosis in arrhythmic MVP. Among AMVPs, the inferolateral segment had the highest ECV% within the LV mid-section. Further, the absolute ECV% in the LV inferolateral segment was significantly greater in AMVP compared to non-AMVP. However, among MVP patients with mild MR, we also highlight a global, multi-segment pattern of interstitial fibrosis which may underline a diffuse myopathic process initially proposed in genetic studies reporting mutations in cardiomyopathy genes (*FLNC, LMNA, ALPK3*A) [38], [39]. A primary myopathy may represent the “substrate” for ventricular arrhythmias in the presence of bileaflet MVP, MAD, and myocardial stretch (the “trigger”). Alternatively, such diffuse myopathy may act alone, when bileaflet MVP and associated imaging arrhythmic features are absent. Indeed, in our study sample, only 50% of MVP patients with mild MR had the traditional phenotype of bileaflet MVP with MAD and curling. Longitudinal studies are needed to determine the prognostic role of CMR-based diffuse interstitial myocardial fibrosis in arrhythmic MVP either as an imaging surrogate of a primary myopathy or, when regionalized to the inferolateral LV, as a precursor of replacement fibrosis.

### 4.2 Sex differences, expansion of myocardial fibrosis, and risk of arrhythmias

There are conflicting findings regarding the potential effect of sex on MVP arrhythmogenicity. Prior reports of MVP-related SCD/SCA cases have shown a predominance of young women [4], [8].

In our study, there was a predominance of women among AMVP cases (60%), consistent with prior observations [40]. Importantly, we observed a stronger association between global ECV% and ventricular arrhythmias in women compared to men. In addition, women with AMVP had significantly greater ECV% in the LV inferolateral segment but also in other segments typically not subject to myocardial traction. The influence of female sex on native myocardial T_1_ and ECV% has been previously documented [41], [42], reinforcing the need for sex-specific cutoffs for T1 mapping parameters. Importantly, when using these sex-specific thresholds for abnormal ECV%, no significant differences were observed between men and women in our study. Furthermore, the distribution of replacement fibrosis as quantified by LGE was also similar between women and men. To our knowledge, this is the first study to investigate and report sex-related differences in the expansion of myocardial fibrosis as measured by CMR in MVP.

Several factors may contribute to sex-specific mechanisms of interstitial fibrosis in AMVP including neurohormonal differences, cardiac structural and functional variations, and genetic predisposition [26], [43]. Sex hormones, particularly estrogen, are well-known for their impact on the myocardium [44]. In this setting, hormonal fluctuations may contribute to ventricular fibrosis and arrhythmias [43]. Greater global/diffuse interstitial fibrosis in women with AMVP and mild MR may suggest a primary/genetic diffuse myopathy with variable, sex-related penetrance similar to that observed for valvular features, i.e., bileaflet MVP and MAD, which are more prevalent in women [45]. Indeed, in addition to greater global ECV%, women with AMVP in our study exhibited greater “regionalized” interstitial fibrosis, specifically higher ECV% in the mid inferolateral LV compared to non-AMVP. Altogether, these findings support the role of sex-specific factors in the development of myocardial fibrosis and subsequent risk of ventricular arrhythmias in MVP. However, further studies are needed to provide a clearer understanding of the effect of sex in arrhythmic MVP.

### 4.3 Clinical implications

The identification of those at highest risk for ventricular arrhythmias and SCD within the larger population of benign MVP cases remains a major and unmet challenge [1]. Current guidelines focus on the management of significant MR, with less emphasis on routine monitoring of ventricular arrhythmias in MVP without MR [46]. In the absence of LGE, arrhythmic risk stratification in MVP is even less clear to the practicing cardiologist. In our study, we demonstrate greater interstitial fibrosis in AMVP, regardless of MR or LGE, and more so in women. Hence, ECV% quantified by T_1_ mapping could be an alternative surrogate marker for myocardial fibrosis that is expected to improve arrhythmic risk stratification in MVP. Individuals with MVP and abnormal ECV%, particularly women without significant MR and/or LGE may benefit from closer clinical and imaging monitoring. Furthermore, our findings support the need for better understanding of the underlying sex-specific mechanisms involved in the pathogenesis of AMVP.

## 5 Limitations

The study’s relatively modest sample size may have limited its statistical power to detect certain associations, particularly the one between AMVP and bileaflet involvement, which could be subject to type II error. The sex-specific thresholds for abnormal ECV% were derived from a relatively small sample size study with a different scanner and imaging protocol. However, recent studies have demonstrated that while native T1 values may vary across different scanners and magnetic field strengths, ECV% remains largely consistent and comparable across various scanners and field strengths [15], [47]. Further studies are needed to corroborate sex-specific thresholds for abnormal ECV%. Although no PVCs were detected during the CMR studies, heart-rate variability remains a possible source of variation in measured T_1_ values [27]. Additionally, elevated T_1_ values could be linked to myocardial edema, as seen in other conditions, rather than fibrosis alone [48]. Future studies incorporating both T_2_ sequences and heart-rate-insensitive sequences or alternative mapping techniques could provide a more accurate assessment of the extent of myocardial fibrosis in AMVP. Monitoring of ventricular arrhythmias was performed during a restricted interval (48 hours), which may not have fully reflected the arrhythmic profile in MVP. Most of the women with AMVP were postmenopausal, which contrasts with the younger age typically reported in SCD cases ascribed to MVP. Nonetheless, it highlights the association between interstitial fibrosis and AMVP in a series of CMR studies conducted in the absence of severe MR. Our findings, which also suggest the first-time sex differences in patterns of fibrosis in MVP and a potential explanation for the higher prevalence of arrhythmic complications in women, need to be validated in further experimental and longitudinal observational studies.

## 6 Conclusion

In the first series of consecutive MVP patients without severe MR, AMVP is associated with greater interstitial fibrosis as quantified by ECV% using CMR/T_1_ mapping, even in the absence of LGE. Measurement of ECV% could help enhance arrhythmic risk stratification, particularly in female AMVP patients, who exhibit greater interstitial fibrosis compared to men, suggesting a diffuse myopathic process. Further studies are needed to determine whether sex-specific mechanisms are involved in the pathogenesis of AMVP.

## Funding

This work was supported by the 10.13039/100000002National Institutes of Health 10.13039/100000050NHLBI R01HL153447.

## Author contributions

**Satoshi Higuchi:** Data curation. **Shafkat Anwar:** Writing—review and editing. **Luca Cristin:** Data curation. **Yoo Jin Lee:** Writing—review and editing, Methodology, Data curation. **Lionel Tastet:** Writing—original draft, Formal analysis, Data curation, Conceptualization. **Henry Hsia:** Writing—review and editing, Data curation. **Rohit Jhawar:** Data curation. **Francesca Delling:** Writing—review and editing, Supervision, Methodology, Funding acquisition, Conceptualization. **Shalini Dixit:** Writing—review and editing. **Mohammad Al-Akchar:** Writing—review and editing, Data curation. **Thuy Nguyen:** Writing—review and editing. **Farzin Arya:** Data curation. **Dwight Bibby:** Data curation.

## Ethics approval and consent

The University of California San Francisco Institutional Review Board approved the study and participants gave informed consent.

## Declaration of competing interests

The authors declare that they have no known competing financial interests or personal relationships that could have appeared to influence the work reported in this paper.

## Data Availability

The data and materials relevant to this study can be obtained from the corresponding author upon reasonable request.

## Footnotes

Appendix A Supplementary data associated with this article can be found in the online version at doi:10.1016/j.jocmr.2024.101117.
