# Supplementary material for: Interstitial fibrosis and arrhythmic mitral valve prolapse: Unraveling sex-based differences
Source: J Cardiovasc Magn Reson. 2024 Oct 28;26(2):101117. doi: 10.1016/j.jocmr.2024.101117 (PMC11652916; doi:10.1016/j.jocmr.2024.101117)
Supplement: Supplementary file 1 — Supplementary material [file mmc1.docx]

**SUPPLEMENTAL MATERIAL**

**Interstitial Fibrosis and Arrhythmic Mitral Valve Prolapse: Unravelling Sex-Based Differences**

Lionel Tastet, Ph.D.^1^; Shalini Dixit, M.D.^1^; Rohit Jhawar, B.A.^1^; Thuy Nguyen, M.D.^1^; Mohammad Al-Akchar, M.D.^2^; Dwight Bibby, RDCS^1^; Farzin Arya, M.D.^1^; Luca Cristin, M.D.^1^; Shafkat Anwar, M.D.^3^; Satoshi Higuchi, M.D.^4^; Henry Hsia, M.D.^4^; Yoo Jin Lee, M.D.^5^; Francesca N. Delling, M.D., M.P.H.^1^

^1^Department of Medicine (Cardiovascular Division), University of California, San Francisco, California, USA

^2^Carle Illinois School of Medicine, University of Illinois-Urbana Champaign, Illinois, USA

^3^Department of Pediatrics, Division of Cardiology, University of California, San Francisco, California, USA

^4^Department of Electrophysiology, Division of Cardiology, University of California, San Francisco, California, USA

^5^Department of Radiology, Division of Cardiology, University of California, San Francisco, California, USA

| **Supplemental Table 1:** Main Characteristics of AMVP Cases with Sudden Cardiac Arrest | | |
| --- | --- | --- |
|  | **Case #1** | **Case #2** |
| *Demographic* |  |  |
| Age, years | 36 | 48 |
| Sex | Female | Female |
| *Morpho-functional characteristics* |  |  |
| MVP subtype | Bileaflet | Bileaflet |
| MR severity | No/trace | Mild |
| MAD inferolateral, n (%) | Yes | Yes |
| MAD inferolateral, mm | 13.0 | 6.4 |
| *LV function and size* |  |  |
| LV ejection fraction, % | 48 | 52 |
| LV mass index, g/m² | 71 | 58 |
| LV systolic curling | Yes | Yes |
| LV systolic curling, mm | 9.4 | 4.2 |
| *Myocardial Fibrosis* |  |  |
| Late gadolinium enhancement | No | Yes |
| Basal inferolateral LV wall | - | Yes |
| Mid inferolateral LV wall | - | - |
| Papillary muscles | - | - |
| LGE size, % | - | 4.0 |
| T_1_ mapping |  |  |
| Basal LV ECV, % | 32 | 39 |
| Mid LV ECV, % | 30 | 32 |
| LGE = late gadolinium enhancement; LV = left ventricular; MAD = mitral annular disjunction; MR = mitral regurgitation; MVP = mitral valve prolapse. | | |

| **Supplemental Table 2:** Factors Associated with the Expansion of Extracellular Volume Fraction | | | | | |
| --- | --- | --- | --- | --- | --- |
|  | **Univariable Analysis** | |  | **Multivariable Analysis** | |
|  | **β ± SE** | **p Value** |  | **β ± SE** | **p Value** |
| ***ECV% Basal Slice*** |  |  |  |  |  |
| Age, years | 0.10 ± 0.04 | 0.44 |  | 0.14 ± 0.04 | 0.25 |
| Female sex | **0.32 ± 1.19** | **0.01** |  | **0.31 ± 1.23** | **0.01** |
| Charlson comorbidity index | -0.01 ± 0.27 | 0.92 |  | - | - |
| PVC origin* | 0.18 ± 1.37 | 0.22 |  | - | - |
| Heart rate, bpm | -0.07 ± 0.05 | 0.58 |  | - | - |
| Hematocrit, % | -0.06 ± 0.17 | 0.66 |  | - | - |
| Bileaflet MVP | 0.17 ± 1.30 | 0.17 |  | 0.17 ± 1.32 | 0.19 |
| MR severity (per grade) | 0.03 ± 0.87 | 0.80 |  | - | - |
| MAD inferolateral ≥6 mm | 0.18 ± 1.30 | 0.15 |  | 0.06 ± 1.32 | 0.65 |
| LV mass index, g/m^2^ | -0.10 ± 0.04 | 0.44 |  | - | - |
| Arrhythmic MVP | **0.35 ± 1.20** | **0.005** |  | 0.22 ± 1.30 | 0.09 |
| ***ECV% Mid Slice*** |  |  |  |  |  |
| Age, years | 0.09 ± 0.04 | 0.47 |  | 0.08 ± 0.04 | 0.49 |
| Female sex | 0.23 ± 1.16 | 0.06 |  | **0.23 ± 1.15** | **0.05** |
| Charlson comorbidity index | -0.03 ± 0.26 | 0.83 |  | - | - |
| PVC origin* | 0.08 ± 1.41 | 0.59 |  | - | - |
| Heart rate, bpm | **-0.32 ± 0.05** | **0.009** |  | **-0.28 ± 0.05** | **0.01** |
| Hematocrit, % | -0.08 ± 0.17 | 0.52 |  | - | - |
| Bileaflet MVP | 0.16 ± 1.22 | 0.19 |  | 0.17 ± 1.18 | 0.16 |
| MR severity (per grade) | 0.03 ± 0.83 | 0.83 |  | - | - |
| MAD inferolateral ≥6 mm | 0.10 ± 0.38 | 0.40 |  | - | - |
| LV mass index, g/m^2^ | -0.02 ± 0.04 | 0.90 |  | - | - |
| Arrhythmic MVP | **0.34 ± 1.15** | **0.006** |  | 0.19 ± 1.21 | 0.13 |
| Values are Beta standardized coefficient (β) ± standard error (SE). Bold numbers indicate statistical significance. *PVC origin: papillary muscles, mitral valve/annulus, or LV/RV outflow tract versus other).  Abbreviations as in **Supplemental Table 1**. | | | | | |

**SUPPLEMENTAL FIGURE 1**


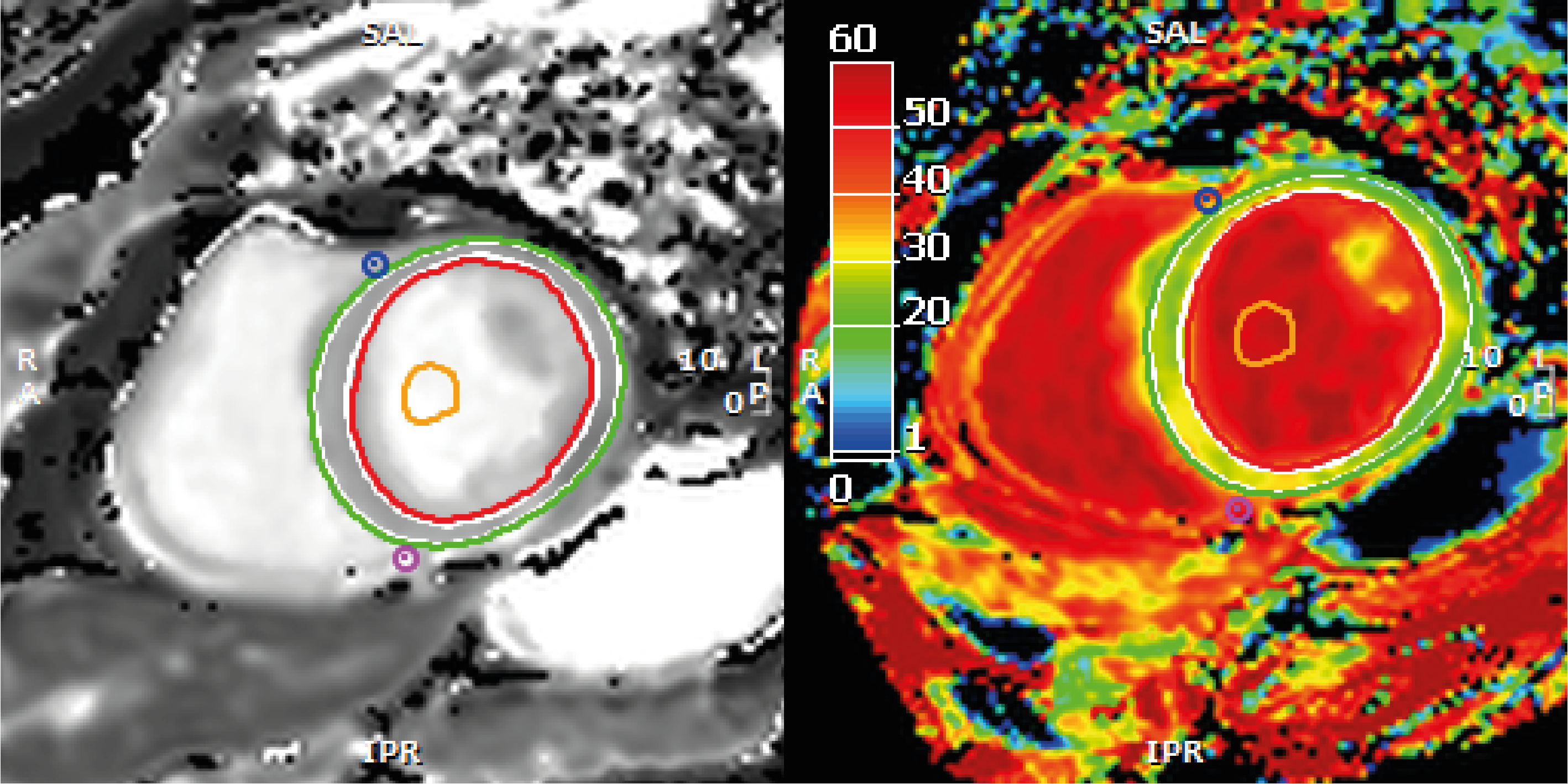


Native T1 map (**left panel**) and extracellular volume (ECV) map (**right panel**) demonstrating manual contouring of the endocardial (**green circle**) and epicardial (**red circle**) borders. To avoid signal contamination from adjacent structures, a 10% inward offset from the endocardial border and a 10% outward offset from the epicardial border were applied (indicated by the **white circle**). This method helps reduce interference from the blood pool and epicardial fat, ensuring more accurate myocardial tissue characterization.
